# ParCuR—A Novel AI-Enabled Gait Cueing Wearable for Patients with Parkinson’s Disease

**DOI:** 10.3390/s25227077

**Published:** 2025-11-20

**Authors:** Telmo Lopes, Manuel Reis Carneiro, Ana Morgadinho, Diogo Reis Carneiro, Mahmoud Tavakoli

**Affiliations:** 1 Institute of Systems and Robotics, Department of Electrical and Computer Engineering, University of Coimbra, 3030-290 Coimbra, Portugal; 2 Department of Health Sciences and Technology, ETH Zurich, 8092 Zurich, Switzerland; manuel.reiscarneiro@hest.ethz.ch; 3 Neurology Department, Centro Hospitalar e Universitário de Coimbra, 3004-561 Coimbra, Portugal; 4 Faculty of Medicine, University of Coimbra, 3000-548 Coimbra, Portugal

**Keywords:** assistive cueing, freezing of gait, machine learning, Parkinson’s disease, somatosensory stimulation, wearable electronics

## Abstract

Freezing of gait (FoG) is a common motor symptom in advanced Parkinson’s disease, leading to falls, disability, and reduced quality of life. Although cueing systems using visual or auditory stimuli can help patients resume walking, existing solutions are often expensive, uncomfortable, and conspicuous. ParCuR (Parkinson Cueing and Rehabilitation) is a compact, ankle-worn wearable integrating an inertial sensor, haptic stimulator, and AI-based software. It was developed to detect FoG episodes in real time and provides automatic sensory cues to assist patients with Parkinson’s Disease (PwP). A classifier was trained for FoG detection using the DAPHNet dataset, comparing patient-specific and patient-independent models. While a small-scale trial with PwP assessed usability and reliability. ParCuR is watch-sized (35 × 41 mm), discreet, and comfortable for daily use. The online detection algorithm triggers stimulation within 0.7 s of episode onset and achieves 94.9% sensitivity and 91.3% specificity using only 14 frequency-based features. Preliminary trials confirmed device feasibility and guided design refinements. This low-cost, wearable solution supports personalized, real-time FoG detection and responsive cueing, improving patient mobility while minimizing discomfort and continuous stimulation habituation.

## 1. Introduction

Parkinson’s disease (PD) is a neurodegenerative disorder that results from the loss of neurons in the substantia nigra pars compacta and leads to reduced striatal dopamine levels [1,2]. Apart from the classic triad of bradykinesia, rigidity, and tremor, one frequently found symptom in advanced PD is Freezing of Gait (FoG). Freezing of gait is characterized by episodes of abrupt and often inexplicable inability to move feet forward, despite their intention to walk, and may affect approximately half of patients with PD (PwP) during the course of the disease [3,4]. As pharmacological treatments often prove ineffective, gait cueing emerges as a valuable therapeutic option [5,6]. Cueing involves the provision of temporal or spatial stimuli aimed at facilitating movement [7].

The cueing goal is immediate FoG mitigation by compensating the loss of movement automaticity and redirecting normal motor control to a goal-directed movement [8,9]. External cueing strategies resource to auditory, visual, and tactile senses and have been shown effective in case of gait impairments such as in stride length, cadence, and FoG [8,10]. One problem that arises is when and how to deliver such stimuli to avoid additional distress in the daily routine of PwP [11,12].

Wearables, encompassing a range of electronic devices worn on the body, may play a pivotal role [13,14,15,16]. These can include sensors and actuators to both monitor changes in physiological movement patterns and respond accordingly. For instance, accelerometer data can be used to detect FoG together with the implementation of an algorithm allowing it to trigger cueing [17,18]. The AI-based algorithms allow for the detection of specific patterns, as motor impairments express high variability [19].

Further than directly improving gait and reducing fall risk, a wearable device can transmit a sense of security by alleviating psychological symptoms, including gait-associated anxiety that may ultimately lead to social stigma, low self-esteem, and even depression [20,21]. Additionally, the continuously recorded data can be used to monitor disease progression and therapy success over time [22,23].

ParCuR (Parkinson Cueing and Rehabilitation) is a miniaturized wearable device capable of detecting and quantifying FoG events and automatically delivering sensory stimulation during such events. This work aimed to develop ParCuR and to perform a preliminary evaluation in PwP with episodes of FoG.

## 2. Materials and Methods

In this work, we created ParCuR and conducted preliminary tests in the miniaturized wearable. This includes the design of a wireless gait monitoring and cueing device, the development of an AI algorithm for the detection of FoG events and stimuli deployment, and a small-scale trial for system validation and user experience analysis. The use of automatically deployed stimuli just for the duration of a FoG event can address the potential issue of habituation to continuous cueing [24].

Critical aspects that were considered when developing ParCuR include its small dimensions (35 × 41 mm), low cost, and durability, which enable comfortable everyday usage and ensure discreet, user-friendly wearability tailored to PwP needs. Moreover, the use of somatosensory cueing was found to be the best fit for this device as it allows the sensor and actuator to be close together in the lower limbs, integrated or hidden by garments with the advantage of the wearable not interfering with the senses of sight and hearing, in contrast to more conventional cueing strategies [12,25,26].

Finally, the wearable was tested in PwP selected from the Movement Disorders Clinic of the Neurology Department of Centro Hospitalar e Universitário de Coimbra to verify usability and assess algorithm performance and cueing efficacy. An experimental setup was planned to induce FoG in the subjects. Questionnaires were applied to assess the impact of the device.

### 2.1. System Architecture and Device Implementation

ParCuR was designed to provide sensory cueing feedback to PwP upon detecting FoG. It accomplishes this by tracking the user’s movements through an Inertial Measurement Unit (IMU) and then transmitting the acquired acceleration data in real-time to a nearby computer. The computer processes the data to detect FoG episodes, and, when these occur, the haptic cueing stimulus is triggered. This stimulus is applied in the patient’s right foot arch to increase sensitivity and reduce discomfort.

#### 2.1.1. Hardware

**Figure 1:**
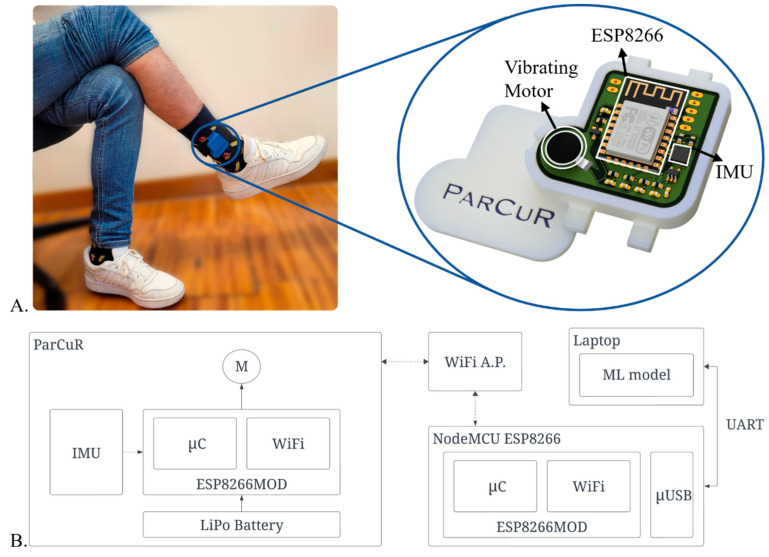
(**A**) Subject wearing the ParCuR device and render with labeled components, including a MPU-6050 for acquiring three axes of acceleration data, with each axis being sampled through a 16 bit ADC, thus, allowing a 8192 LSB/g accelerometer sensitivity for a ±4 g full-scale range, an ESP8266 microcontroller with built-in Wi-Fi communication for wireless data transfer, and a haptic actuator (vibration motor) for delivering the cueing stimulus; (**B**) Block diagram of system implementation. Communication is established between ParCuR and the NodeMCU ESP8266 over a Wi-Fi access point (WiFi A.P.). Following, the computer where the ML model and a graphic interface runs, is connected via USB port to the NodeMCU ESP8266. In the ParCuR wearable device, IMU data is acquired by the microcontroller (µC) and send for evaluation by the ML model. After receiving the model feedback, the ParCuR microcontroller will activate or disactivate the vibrating motor (M).

To meet the design goals of creating a discreet, real-time FoG detection device with a small form factor for concealed wear, state-of-the-art microchips were integrated into a Printed Circuit Board (PCB) prototype (Figure S1). The main components of the device, detailed in Figure 1A, include an MPU-6050 (TDK InvenSense, San Jose, CA, USA), an ESP8266 microcontroller (Ai-Thinker Co., Ltd., Shenzhen, China) with built-in Wi-Fi communication for wireless data transfer, and a haptic actuator (vibrational motor (Seeed Technology Co., Ltd., Shenzhen, China)) for delivering the cueing stimulus [27].

In Figure 1A, a subject is wearing the ParCuR device in the desired position for signal acquisition and stimuli delivery. In ParCuR, data from the ESP8266 module will be transmitted via Wi-Fi to a NodeMCU ESP8266 (Joy-IT, Neukirchen-Vluyn, Germany) board connected to the computer through USB serial (Figure 1B). To enable simultaneous acquisition of multiple devices, a WiFi access point is used to manage the network. After processing the IMU’s data, when FoG is detected, an output value is fed through the inverse path—from computer to wearable—to control the vibrational motor, triggering the rhythmic cueing signal. The stimulation follows a rhythmic pattern obtained through the repeated activation and deactivation of the motor. The system integrates a 300 mAh, 3.7 V LiPo battery (Shenzhen Pknergy Energy Co., Ltd., Shenzhen, China). Direct current measurements indicated a consumption of approximately 79.3 mA with the vibration motor off (Wi-Fi connected) and about 151.7 mA when the motor was active. Based on these values, the estimated autonomy ranges from 120 (motor always on) to 225 min (motor always off), depending on motor activity. However, this estimate reflects laboratory conditions, while real-world operation, characterized by frequent stimulation cycles during daily activities, will likely reduce the effective battery life. Future work will assess long-term autonomy and power optimization under realistic usage patterns. The prototype is assembled in a custom 3D-printed case attached to an elastic strap with Velcro (The Creative Club, Schenefeld, Germany) for easy use. In addition to the small footprint, the total weight is 39.60 g and the overall bill of materials is EUR 9.40.

#### 2.1.2. Software

The software system is divided between the wearable—equipped with the ESP8266 for sensor data acquisition, data transfer, and cueing deployment—and an external computer for signal processing and running the Machine Learning (ML) algorithm and communicating with the wearable.

The microcontroller (µC) acquires the IMU at a sampling rate of 64 Hz and a packet containing the sampled data, timestamp, and motor activation status is created and sent to the computer. In addition, return packets with the classifier feedback regarding the occurrence of FoG and the need for cueing deployment are received by the µC. The WiFi network relies on a User Datagram Protocol approach due to the time-sensitive aspect of FoG detection.

A simple graphic interface (Figure S2) was developed and allows the technician or healthcare professional to visualize the signal and control the system in real-time in terms of the desired frequency for the haptic stimulation. The interface shows three moving plots corresponding to the last 1 min acceleration signal of three distinct wearables. An additional plot represents the ML model prediction, indicating normal gait or the presence of FoG. The system is still fully functional in standalone mode with no need for the GUI to be running.

### 2.2. Algorithm Implementation

Before a custom wearable was designed and the data from PwP was acquired, the FoG online detector was trained and validated on the pre-existing DAPHNet dataset by Bächlin et al., 2010 [28].

The publicly available DAPHNet Freezing-of-Gait dataset includes data from 10 PwP (seven males, three females; mean age 66.5 ± 4.8 years) with a documented history of FOG. Each subject wore three triaxial accelerometers placed above the ankle (shank), above the knee (thigh), and on the lower back, generating nine acceleration channels sampled at 64 Hz. The trial comprised three walking tasks to induce FoG: straight walking, walking with frequent turns, and an ADL-style circuit (opening doors, fetching coffee, etc.). Across all recordings, it contains approximately 500 min of continuous acceleration data. Every data sample is time-stamped and labeled with one of three categorical annotations: “0” for non-experimental periods, “1” for movement without freezing, and “2” for movement with FoG. Eight of the ten participants displayed FoG, yielding 237 annotated FoG episodes (0.5–40.5 s).

For analysis, we used only the shank/ankle sensor to match ParCuR’s placement. The continuous recordings were segmented with a 4 s sliding window (256 samples) and a 0.5 s step (32 samples), generating an 87.5% overlap between consecutive windows. This corresponds to ≈60,000 total segments across subjects (((30,000 s − 4 s)/0.5 s + 1) ≈ 59,993).

ParCuR operates from an ankle-mounted IMU; therefore, in DAPHNet we purposely used the shank/ankle channel for all training and tests to match our hardware. The “freezing band” (3–8 Hz) reflects lower-limb trembling and is present at multiple locations but is strongest distally. Prior work shows that a single shank sensor provides performance comparable to multi-sensor systems and superior to hip in many settings, while a lumbar sensor can also be viable.

A 15 Hz low-pass filter was used because human gait dynamics primarily occur below 15 Hz, and frequencies above this range are dominated by sensor noise and high-frequency artifacts unrelated to locomotion. Prior FoG studies using the DAPHNet dataset and similar IMU-based systems have consistently applied cutoff frequency filters [29,30,31]. Filtering at 15 Hz therefore preserves physiologically relevant gait components while attenuating noise, improving the stability and reliability of the extracted features [29,30,31].

A set of time-domain and statistical features frequently applied in activity recognition are proposed to train the supervised learning algorithm [32], as well as frequency-based features. In this work, the frequency-based features fed to the ML algorithm were the Freezing Index (FI)—defined as a ratio between the power in the freezing band (3 to 8 Hz) and the power in the locomotion band (0.5 to 3 Hz) [29], and the frequency and magnitude of the two highest amplitude peaks in the frequency domain of the acceleration, corresponding to the stride and stance frequencies (approximately 1 and 2 Hz, respectively) [30]. The time-domain features are the minimum, maximum, median, and mean amplitude of the signal, its root mean square (RMS) value, standard deviation (STD), kurtosis, skewness, entropy, zero crossing rate (ZCR), Mean Crossing Rate (MCR), and the sum of the magnitudes over the entire window (Table S1 details the considered features).

The available data was divided into train and test sets to fit the ML model and evaluate its fit, respectively. Two splitting techniques were used to analyze the dataset. The first technique, Leave-One-Patient-Out (LOPO), uses all patients but one to train the classifier, and the remaining one to test it. The LOPO method creates a generic model as the used signals come from multiple patients. However, FoG patterns can change from patient to patient, and the model can fail to make correct predictions. Conversely, the K-Fold Cross-Validation (K-fold) model is trained and tested with data obtained from a unique user, achieving better classifications for that specific patient with less training data by capturing more patient-specific characteristics [31,33]. To address the unbalanced labeling caused by the fact that normal gait events occur much more often than FoG episodes, the *RepeatedStratifiedKFold* function from the scikit-learn library splits 75% of normal and FoG-related features to training procedures and the remaining for testing, hence ensuring not only the presence but the balance of FoG episodes in both training and testing sets.

We selected a Support Vector Machine (SVM, RBF kernel) as our primary classifier since we are dealing with a small-to-moderate dataset (10 subjects with a strong class imbalance), a setting where margin-based methods typically generalize well with limited overfitting. Moreover, SVMs are effective in high-dimensional spaces without requiring large training sets, and the trained model has a very small memory footprint and yields fast, constant-time predictions—compatible with our real-time (<16 ms pipeline) and, in future iterations, compatible with embedded constraints. These factors—together with our need for a compute- and memory-efficient model that can, in future works be deployed directly on a low-power microcontroller—led us to prioritize SVM over more complex deep models or large ensembles.

The classification stage was performed using SVM implements with the scikit-learn library in Python 3.10. To ensure optimal model performance, an internal optimization routine was included to automatically tune the SVM hyperparameters by performing a grid search with cross-validation using the *GridSearchCV* method. This method explored several values for the regularization parameter (C ∈ {0.01, 0.1, 1, 10, 100}) and kernel coefficient (γ ∈ {0.001, 0.01, 0.1, 1}). For each combination, a k-fold cross validation (k = 5) was applied to the trained data, and the mean F1 score across folds was computed to serve as optimization criterion. The final SVM model was then retrained on the entire training dataset using the optimal parameters before being evaluated on the test data.

### 2.3. Experimental Setup

ParCuR was tested in a small-scale trial with PwP recruited from the Movement Disorders Clinic of the Neurology Department of Centro Hospitalar e Universitário de Coimbra. This trial assessed the detection and cueing efficacy in FoG mitigation as well as the usability and efficacy of the developed wearable. Patients were enrolled based on their capabilities to cooperate with the proposed tasks, preserved ability to walk without physical help from someone despite episodes of FoG, adequate hearing and vision capacities, and absence of other disorders that might interfere with gait. For a matter of comfort and to increase the efficacy of signal capture, the wearable was placed on the right ankle of each patient, and the sensor was placed in the sole.

#### Experimental Protocol

**Figure 2:**
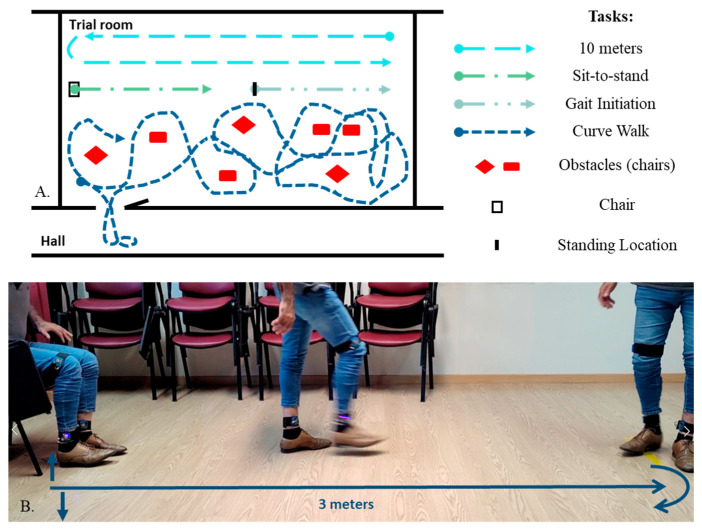
Scheme of the tasks accomplished in the experimental procedures. (**A**) 10 m, sit-to-stand, gait initiation, curve walk tasks; (**B**) TUG test.

Each trial was divided into two sessions. The first session was used for signal acquisition from the patient during various tasks, as well as testing cueing stimulus deployment with a generic firmware trained with the DAPHNet dataset. For the second session, a similar signal acquisition took place, in addition to testing the wearable cueing strategy with a patient-specific FoG detection algorithm trained on the previously acquired data. For evaluation of the overall wearable performance and assessment of the efficacy of on-demand cueing vs. continuous stimulation, two additional testing scenarios were included in the trial: continuous haptic stimulation provided by the wearable during the motor tasks, and continuous metronome-based auditory cueing during the various tasks. Each session is divided into stages (Table S2) where the same motor protocol is performed in different experimental conditions. The motor protocol included six tasks (Figure 2) chosen to analyze gait aspects and to provoke a FoG episode. Video recordings of the tasks were used to evaluate gait and label FoG episodes by an experienced clinician.

At the end of each session, participants filled out a questionnaire (Note S3) with questions related to FoG events, their impact on daily life, and the usability, simplicity, comfort, and effectiveness of the implemented wearable cueing device. Comfort Rating Scales (CRS) were employed to assess the comfort of the user [34].

## 3. Results

### 3.1. Detecting Freezing of Gait Episodes Using the DAPHNet Dataset

**Figure 3:**
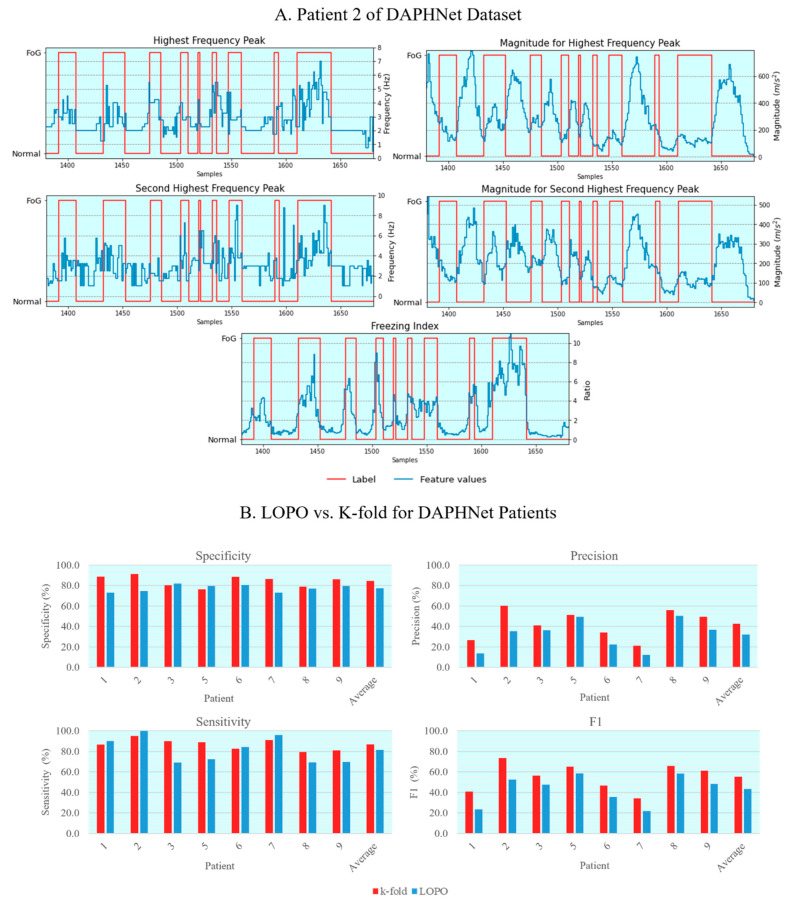
(**A**) Performance of three frequency-based features (blue) extracted from DAPHNet patient 2 on the right axis. These feature values were obtained from 4 s windows during 5 min of recorded signal. In the left side axis are represented the FoG specialist determined labels (FoG or Normal) given by the dataset. These graphics illustrate the descriptive power of the represented features, since FoG events overlap with higher frequency and lower magnitude on the highest frequency peak when compared with normal walking. Also, as expected, the Freezing Index displays higher ratios during FoG events; (**B**) Comparison of classification metrics for the LOPO and K-fold methods using a SVM classifier. These results were obtained for the eight patients of DAPHNet dataset that demonstrated freezing. Overall, the K-fold method consistently top ranks the LOPO approach, highlighting the superior performance of a model trained specifically for individual patients.

To test whether the developed algorithm was able to detect FoG events the DAPHNet dataset was used. Figure 3A shows the relation between the previously discussed frequency-domain features derived from the acceleration magnitude signal, and the presence or not of FoG events. This data corresponds to 5 min recording of the same signal for comparison purposes. For the Highest (HFP) and Second Highest (SHFP) Frequency Peak features, the values tend to rise during FoG and decrease for normal gait, thus confirming a signal shift for higher frequencies during an episode of FoG. In addition, the Magnitudes of these peaks (MHFP and MSHFP) declined most of the time during FoG, which denoted a lower level of activity and decreased movement amplitude. The frequency shift and magnitude decrease can be unnoticeable when other disorders impact movement or the patient displays more violent FoG episodes. Finally, the FI appears to be a good indicator of FoG episodes as it increases rapidly when FoG starts, maintaining a high magnitude until normal gait is resumed. All five features have the potential to identify FoG, although the SHFP and MSHFP seem to be a downgraded version of the HFP and MHFP, respectively.

The classification results are described and analyzed based on the two methods—LOPO and K-fold—used to split the dataset into training and testing sets. The features corresponding to RMS, Mean, and STD were discarded (Note S4). The performance evaluation was accomplished by comparing the dataset label with the model prediction in the test set.

The classification results of the LOPO method used to train an SVM classifier in a universe of eight patients are displayed in parallel with the K-fold method in Figure 3B (Table S3). Regarding LOPO, patient 2 stands out for the maximum sensitivity (100%), meaning that the model detected all episodes of FoG in that patient. However, the specificity value (74.7%) is much lower since many normal gait windows were considered FoG. These results emphasize the trade-off around detection: more true FoG detections will also imply more false positives. As can be seen, a personalized model fitted to each patient would allow better recognition of individual freezing patterns and, by decreasing the scope of what can be classified as FoG, avoid wrong classifications.

In Figure 3B (Table S4), the method implemented is patient specific with the train and test data coming from the same patient. The classification results were obtained with the same SVM classifier, but with a K-fold Cross Validation method. In addition to the metrics used to evaluate the model output, prevalence is included in Table S4 as a method to quantify freezing in the patient’s sets since it explains the ratio between the number of windows with freezing and the total number of signal windows. Taking patient 2 as an example, 94.9% of freezing windows were correctly identified and 60.2% of the identified freezing is actually freezing. Also, the specificity (91.3%) and F1 score (73.7%) display good result values.

In general, all patients scored better with the patient-specific-train method in most of the metrics used. For the LOPO method, averaging all patients’ models results, the specificity reached 77.5 ± 2.9% and the sensitivity 81.4 ± 11.2%. While for the K-fold the specificity and sensitivity were 84.6 ± 4.5% and 86.8 ± 4.4%, respectively. It is possible to conclude that individually trained methods are superior to generalized methods in FoG classification problems. The entire pipeline for FoG detection (pre-processing, segmentation, feature extraction, and classification) takes a maximum of 15.63 milliseconds to complete, which makes it extremely efficient for real-time FoG detection. Moreover, the time lasting from the episode onset until the stimulus is activated does not exceed 0.7 s.

**Figure 4:**
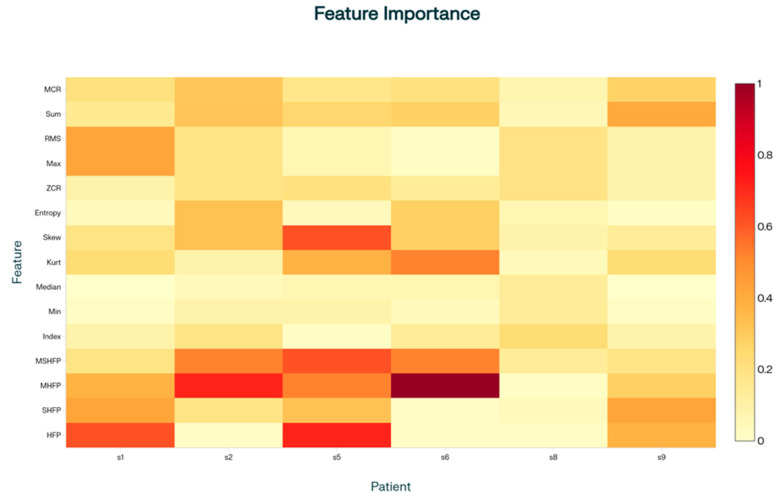
Normalized feature importance values (red to yellow) indicate the relative contribution of each feature to FoG detection per patient using k-fold model.

The feature importance values shown in Figure 4 correspond to the application of a k-fold model individually to each patient exhibiting FoG. For FoG detection, the most important features are those capturing signal irregularity, variability, and sudden changes, with a particular focus on medium and high frequency components (MHFP and MSHFP), metrics such as skewness and kurtosis, entropy, and signal intensity (RMS and Max). These features are more discriminative because they reflect the complex and abrupt motor phenomena typical of FoG episodes.

### 3.2. Testing the System on Human Subjects

**Figure 5:**
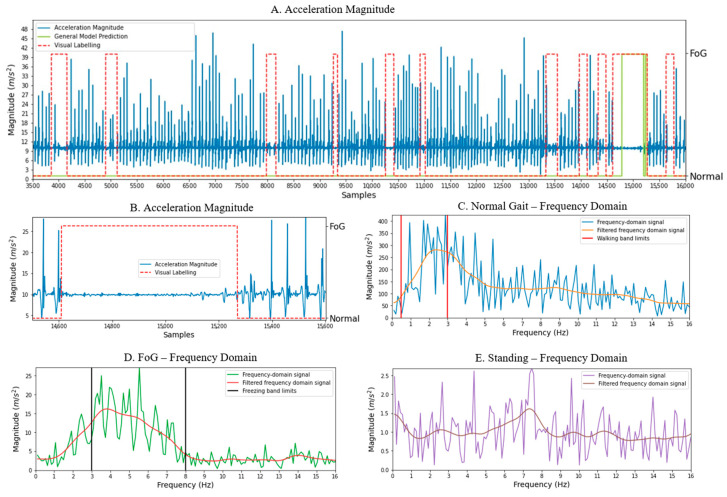
(**A**) Acceleration magnitude signal with overlap FoG events obtained from the general ML model and visual labeling based on the videotapes; (**B**) Acceleration magnitude signal—Normal and FoG—with overlap visual labeling; (**C**–**E**). Frequency-domain signal calculated from 600 samples excerpts of Normal Gait, FoG and Standing states, accordingly. The vertical lines mark the limits of the freezing and walking bands proposed by Moore et al. [14]. All excerpts were removed from the Curve walk test—Stage 2.3 of patient 2. As an example, it is noticeable the peak frequency shift for a higher band of frequencies during FoG, although with lower magnitude. Also, comparing FoG and Standing states, the frequency peaks show one order of magnitude difference.

At the moment of the evaluation, patient 1 was 65 years old, had PD for 11 years, an MDS-UPDRS-III score of 15, and a modified H&Y of 2. He showed upper and lower limb severe resting tremor. There were no motor or sensory disabilities that inhibited the experiments or any signs of initial fatigue. Patient 2 was 73 years old and had an akinetic-rigid form of PD for 7 years, an MDS-UPDRS-III score of 20, and a modified H&Y of 2.5. Neither patient experienced an incident of FoG during their first session. Although this prevents the patient-specific detection algorithm from working properly, we proceeded to the second session to deploy the cueing strategy. In the second session, patient 1 completed all tasks without FoG. Patient 2 had one cluster of consecutive episodes of FoG occurring over an approximately 10 m path. This patient took 1 min and 51 s to travel (average speed of 0.090 m/s) the distance, a value that contrasts with the mean velocity of 1154 m/s for individuals over 71 years old [35]. In Figure 5A, an excerpt of the task illustrates eleven FoG events (red) with an average duration of 3.32 s per episode. The general model was able to detect FoG corresponding to a longer-duration episode. In the FoG portion of Figure 5B, small pulses are visually noticeable in place of the normal gait cycles. These pulses seem to be characteristic in FoG episodes of patient 2 since standing periods of the same patient did not produce them.

In Figure 5, we addressed three possible scenarios—normal gait, FoG, and standing—with plots representing the frequency domain of 600 samples (9.38 s) excerpts of normal gait, FoG, and standing signals from patient 2. It is perceptible that normal gait and FoG signals have, respectively, the highest frequency peak in the walking band and freezing band limits (Figure 5C,E). During FoG, spectral power shifts toward the 3–8 Hz freezing band and overall magnitude decreases relative to normal gait. In standing, no clear peaks are present in either locomotion or freezing bands, and power remains near the noise floor. (Figure 5E).

Regarding the first questionnaire, both patients stated that FoG has a moderate to severe impact on their daily lives and the two already had falls and need for medical care because of FoG (Table S5). Regarding the second questionnaire, patient 1 reported no prior familiarity with assistive devices for FoG and indicated that he would be more inclined to use the system if it were not visible, mainly due to concerns about stigma. He also highlighted the importance of the on-demand stimulation mode, noting that continuous cueing can become disruptive during daily living. Patient 2 was incapable of completing the second questionnaire due to fatigue and OFF-related anxiety at the end of the second session. Patient 1 considered the wearable as discrete, safe, unobstructive, reliable, and nearly an extension of the human body (Figure S3). However, he had concerns related to the device’s presence on the body and the employed vibration being potentially disturbing in long-term use.

The feasibility sessions yielded very few FoG events (P1: 0; P2: 1). Consequently, conventional window-level metrics (accuracy, specificity, F1-score) are not statistically interpretable in this context and are therefore not reported. These metrics become dominated by true-negative windows and are unstable or undefined when the number of positive events is ≤1, further compounded by the lack of independence introduced by overlapping windows. For this reason, quantitative performance is reported only in Section 3.1 (DAPHNet), while Section 3.2 is dedicated to feasibility and user feedback. A larger, adequately powered study will be required to estimate accuracy-based metrics during real-world use.

## 4. Discussion

Freezing of gait is a poorly treated symptom of advanced PD. We developed ParCuR, a comfortable wearable with inertial data collection, wireless communication, and haptic stimulation capabilities. An online FoG detection algorithm was created using supervised learning and tested with a reduced feature set (14 features) that contrasts with the larger number of features used in previous works (55 features in the work of Rodríguez-Martín and colleagues [31], demonstrating effective FoG detection with low latency (stimulus activation within 0.7 s from episode onset).

Compared with previous FoG detection studies, our system achieves superior performance while using a single ankle-mounted IMU and a compact feature set. As summarized in Table 1, earlier threshold-based approaches reported sensitivities between 73 and 89%, and machine-learning methods such as decision trees [32], SVMs [31], or CNNs [36] typically required larger feature sets, multiple sensors, or offline processing. In contrast, this study reaches 94.9% sensitivity and 91.3% specificity with a 4 s window, 0.7 s latency, and only 14 frequency-based features, demonstrating that a lightweight, real-time pipeline can match or exceed the accuracy of more complex or multi-sensor systems.

The classification developed after the DAPHNet dataset showed good detection capabilities. For the LOPO method, on average, the specificity reached 77.5 ± 2.9% and the sensitivity 81.4 ± 11.2%. For the K-fold method, the results are better than the last as expected although more patient-specific. On average the specificity and sensitivity are 84.6 ± 4.5% and 86.8 ± 4.4%, respectively. We believe that it is worth training the device to the patient and thus improving its detection efficacy to reach the goal of daily use of the wearable.

Although FI-based features may transfer across sensor locations, in this work we matched training and deployment to the ankle to avoid potential domain shift. Future studies will explicitly quantify cross-location generalization (e.g., ankle vs. thigh/lumbar) under the proposed pipeline.

In contrast to most existing devices that are visible and may cause stigma, our wearable and haptic stimulator are small and can be easily concealed under clothing, mitigating this issue. The option for on-demand triggering enhances user comfort and reduces habituation to continuous stimulation. Also, this work showed the importance of reducing the vibration period to the minimum possible time by activating it only when required to attenuate FoG episodes.

In the context of developing a wearable device for FoG detection and gait cueing, this system stands out for its cost-effectiveness, wearability, and discreet profile beneath clothing without compromising performance. However, the lack of extended home use remains a key limitation; a prospective multi-week assessment is planned to capture adherence, habituation, and social acceptability. While this work represents an initial step in addressing FoG, several challenges remain. Future research concerning the device should focus on enhancing the wearable’s functionality, validating its efficacy in clinical settings, and streamlining its data processing by integrating the entire pipeline within the device. This integration could eliminate the need for an external processor (e.g., a computer) and reduce communication delays, possibly by using an upgraded microcontroller. Additionally, firmware improvements should target reduced power consumption to extend the device’s two-hour battery life and decrease the battery’s size and weight.

Efforts should also investigate methods for reducing the vibration motor’s energy consumption. Usability studies are needed to determine the minimal G-force required for users to perceive stimulation successfully, potentially by driving vibration motors at lower voltages. However, this may come at the cost of reduced motor speed and vibration force. Alternatively, more efficient, albeit pricier, low-consumption motors are available on the market. While not evaluated in the present study, transcutaneous electrical stimulation offers an avenue for lowering energy consumption. Placing the stimulus in the midfoot maintains a sufficient distance from the ankle’s inertial sensor to allow uninterrupted data collection during stimulation. This location is chosen for its high sensitivity, although further research should optimize sensitivity across various body locations while maintaining the benefits of sensory cueing in specific anatomical regions. The cost of the device can be significantly reduced by mass production, which would also lead to the widespread adoption of this wearable.

Addressing the coexistence of the inertial sensor and vibration motor within the compact wearable device presents a challenge. Potential solutions include isolating the stimulus using damping materials, with the caveat that the human body may act as a mechanical conductor between the stimulus and the sensor. Other options involve temporarily disabling data collection during stimulus activation or employing sensory electrical stimulation.

We performed a pilot study in two PwP who had their gait improved with haptic stimulation when wearing ParCuR. The performed experiments have, once again, shown the importance of paying close attention to the issues of habituation and stigma from visible wearables, when designing such a system. We believe that a large-scale trial is required to fully assess wearable usability, online FoG detection capabilities, and the effectiveness of haptic cueing to ultimately establish ParCuR as a valid option for dealing with FoG. Lack of extended home use is a key limitation; a prospective, multi-week assessment is planned to capture adherence, habituation, and social acceptability. Regarding the questionnaires and further dialog with the patients, they seemed to be likely users of the wearable once it proved to enhance walking and be not visible. During the whole experience course, patients described the wearable as comfortable and easy to wear in everyday life. Although the small sample, the experiments performed, and the invaluable patient input have supported the existence of issues such as habituation and stigma from visible wearables that the ParCuR wearable may overcome soon.

## Figures and Tables

**Table 1:** Summary of FoG detection algorithms reported in published studies.

Author	Year	Patients	Sensors	Location	Methods	Contributions		Sliding Window	Sampling Freq.	Delay
Moore et al. [29]	2008	11	1 IMU	Left Ankle	Threshold-based (FI)	Introduction of FI Generic threshold detected 78.3% Patient adapted threshold detected 89.1%		6 s	100 Hz	(Offline analysis)
Bächlin et al. [28]	2010	10	1 IMU	Left Ankle	Threshold-based (FI&PI)	Introduction of PI Online detection with generic thresholds Sensitivity: 73.1%; Specificity: 81.6%		4 s Step: 0.5 s	64 Hz	Max. 2 s
Moore et al. [30]	2013	25	7 IMU	Lower back, ankles, feet and knees	Threshold-based FI	Evaluation of different sensor locations, windows size and threshold values	Sensitivity: 84.3% Specificity: 78.4%	7.5 s	50 Hz	(Offline analysis)
			1 IMU	Left or Right Ankle			Sensitivity: 86.2% Specificity: 66.7%			
Mazilu et al. [32]	2014	5	2 IMU	Ankles	Decision tree classifier	Detected: 99 of 102 real episodes and 27 false alarms Sensitivity: 97%		2 s Step: 0.25 s	32 Hz	Max. 0.5 s
Rodríguez-Martín et al. [31]	2017	21	1 IMU	Waist	SVM	Generic model—not adapted to any patient Sensitivity: 74.7%; Specificity: 79.0%		3.2 s Step: 1.6 s	40 Hz	(Offline analysis)
						Personalised model–trained per patient Sensitivity: 88.1%; Specificity: 80.1%				
O’Day et al. [36]	2022	7	1 IMU	Ankle	CNN	AUROC *: 0.80 Precision: 61%		2 s	64 Hz	(Offline analysis)
This study	-	10	1 IMU	Ankle	SVM	14 frequency-based features Sensitivity: 94.9%; Specificity: 91.3%		4 s Step: 0.5 s	64 Hz	0.7 s

* AUROC—Area Under the Receiver Operating Characteristic. The maximum value being 1 means that the diagnostic test is perfect in the differentiation between the diseased and no diseased. The value 0.5 should be the minimum since that represents the chance level.

## Data Availability

Publicly available datasets were analyzed in this study. This data can be found here: [https://archive.ics.uci.edu/static/public/245/daphnet+freezing+of+gait.zip (accessed on 21 October 2025)]. The data that supports the findings of this study are available from the corresponding authors upon reasonable request.

## Supplementary Materials

The following supporting information can be downloaded at: https://www.mdpi.com/article/10.3390/s25227077/s1, Figure S1: ParCuR Printed Circuit Board; Figure S2: ParCuR Graphical User Interface; Figure S3: CRS results from questionnaire 2; Figure S4: Features selected; Table S1: Time domain and frequency features extracted from the DAPHNet dataset; Table S2: Experimental study organization for testing the wearable; Table S3: LOPO results; Table S4: K-fold results; Table S5: Results from questionnaire 1; Table S6: Summary of subjects’ information at the time of data acquisition for the DAPHNet dataset; Note S1: Detailed description of the DAPHNet dataset; Note S2: Detailed description of the signal processing pipeline for the DAPHNet data; Note S3: Questionnaires filled by volunteers in the trial; Note S4: Discussion on feature selection.

## Abbreviations

The following abbreviations are used in this manuscript:

FOG	Freezing of Gait
ParCuR	Parkinson Cueing and Rehabilitation
PwP	Patients with Parkinson
PD	Parkinson’s Disease
IMU	Inertial Measurement Unit
PCB	Printed Circuit Board
ML	Machine Learning
µC	Microcontroller
ADL	Activities of Daily Living
FI	Freezing Index
RMS	Root Mean Square
STD	Standard Deviation
ZCR	Zero Crossing Rate
MCR	Mean Crossing Rate
LOPO	Leave-One-Patient-Out
K-fold	K-Fold Cross-Validation
SVM	Support Vector Machines
CRS	Comfort Rating Scales
HFP	Highest Frequency Peak
SHFP	Second Highest Frequency Peak
MHFP	Magnitude of Highest Frequency Peak
MSHFP	Magnitude of Second Highest Frequency Peak
