# Supplementary material for: ParCuR—A Novel AI-Enabled Gait Cueing Wearable for Patients with Parkinson’s Disease
Source: Sensors (Basel). 2025 Nov 20;25(22):7077. doi: 10.3390/s25227077 (PMC12656022; doi:10.3390/s25227077)
Supplement: Supplementary file 1 [file sensors-25-07077-s001.zip › sensors-3969940-supplementary.pdf]

Supplementary Materials

ParCuR—A Novel AI-Enabled Gait Cueing Wearable for Patients with Parkinson's Disease

Telmo Lopes, Manuel Reis Carneiro, Ana Morgadinho, Diogo Reis Carneiro, and Mahmoud Tavakoli

Figure S1: A. ParCuR schematic; B. ParCuR PCB layout on Eagle software with the associated measures.
Figure S2: Illustration of the developed graphical user interface.
Figure S3: Comfort assessment of the wearable device by patient 1. The scales used – CRS – range from 0 to 20, respectively, low and high.
Figure S4 - Number of times that each feature was selected by the Sequential Feature Selector. Frequency-based features: HFP - Highest Frequency Peak, SHFP - Second Highest Frequency Peak, MHFP - Magnitude for Highest Frequency Peak, MSHFP - Magnitude for Second Highest Frequency Peak, Index - Freezing Index. Time-domain featured: Min – Minimum of magnitude signal, Max – Maximum of magnitude signal, Median, Mean, RMS – Root Mean Square, STD – Standard Deviation, Kurt- Kurtosis, Skew – Skewness, Entropy, ZCR – Zero Crossing Rate, MCR – Mean Crossing Rate, Sum – sum of magnitude values.
Table S1: Group of frequency-based features and time-domain and statistical features used in FoG detection algorithm their description and purpose in gait characterization.
Table S2: Experimental study organization in two sessions with different stages. Motor protocol is repeated for all stages, each stage includes at least one variable change.
Table S3: Classification results of the testing set using the LOPO method for the eight patients of DAPHNet dataset that demonstrates freezing. The 'Test Patient' represents the set used to test the classifier trained with the remaining seven patients' data, using a SVM classifier.
Table S4: Classification results of the testing set for the eight patients of DAPHNet dataset that demonstrates freezing. The results were obtained through Repeated Stratified k-fold Cross Validation method for each patient using a SVM classifier. In addition to the metrics used to evaluate the classification, prevalence explains the ratio between the number of windows with freezing and the total number of signal windows. The values are in percentage.
Table S5: Resume of questionnaire 1 important aspects.
Table S6: Summary of subjects' information at the time of data acquisition for the DAPHNet dataset.

Note S1: Detailed description of the DAPHNet dataset
Note S2: Detailed description of the signal processing pipeline for the DAPHNet data
Note S3: Questionnaires filled by volunteers in the trial
Note S4: Discussion on feature selection

1 Supplementary Figures

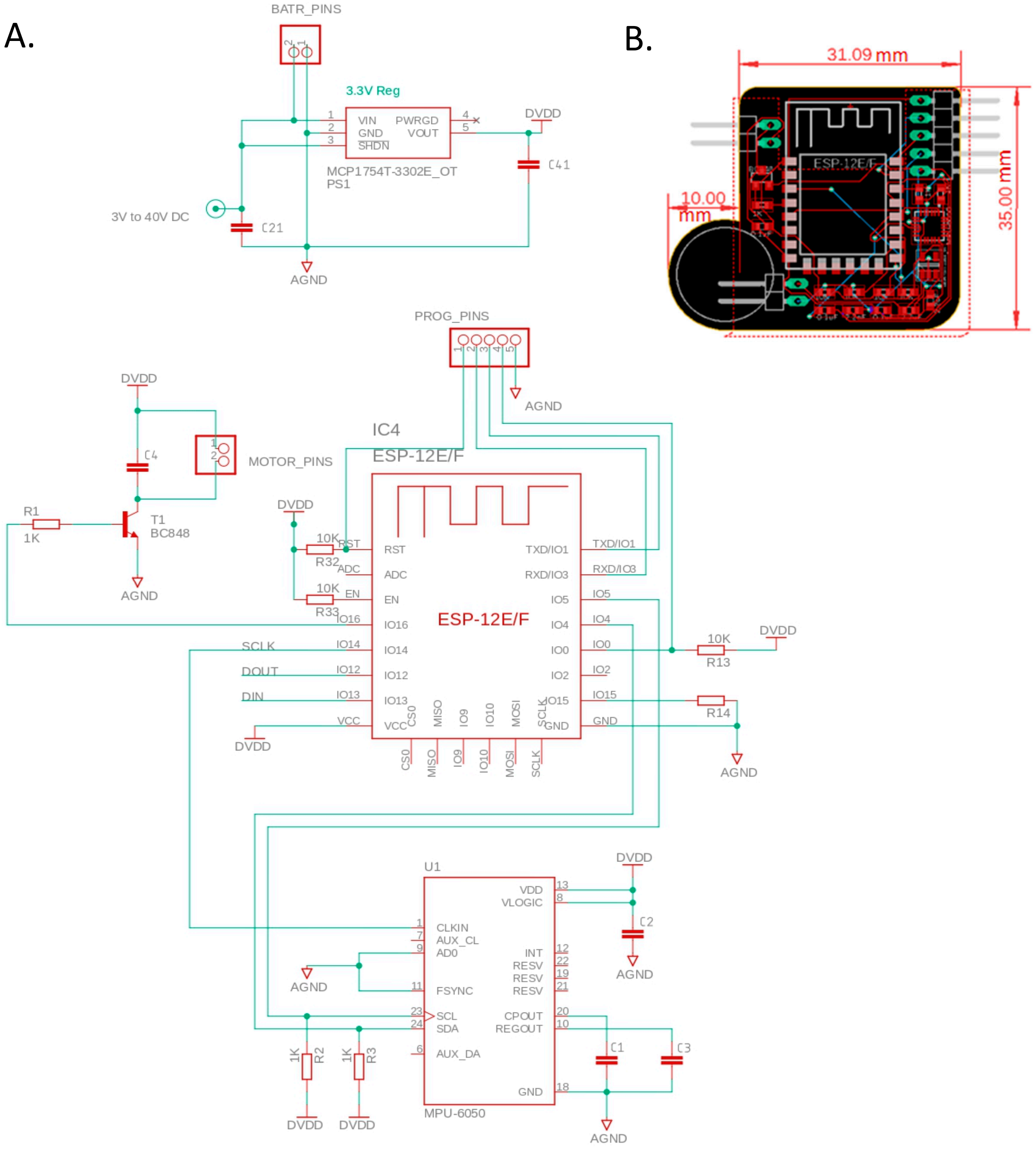

Figure S1 - A. ParCuR schematic; B. ParCuR PCB layout on Eagle software with the associated measures.

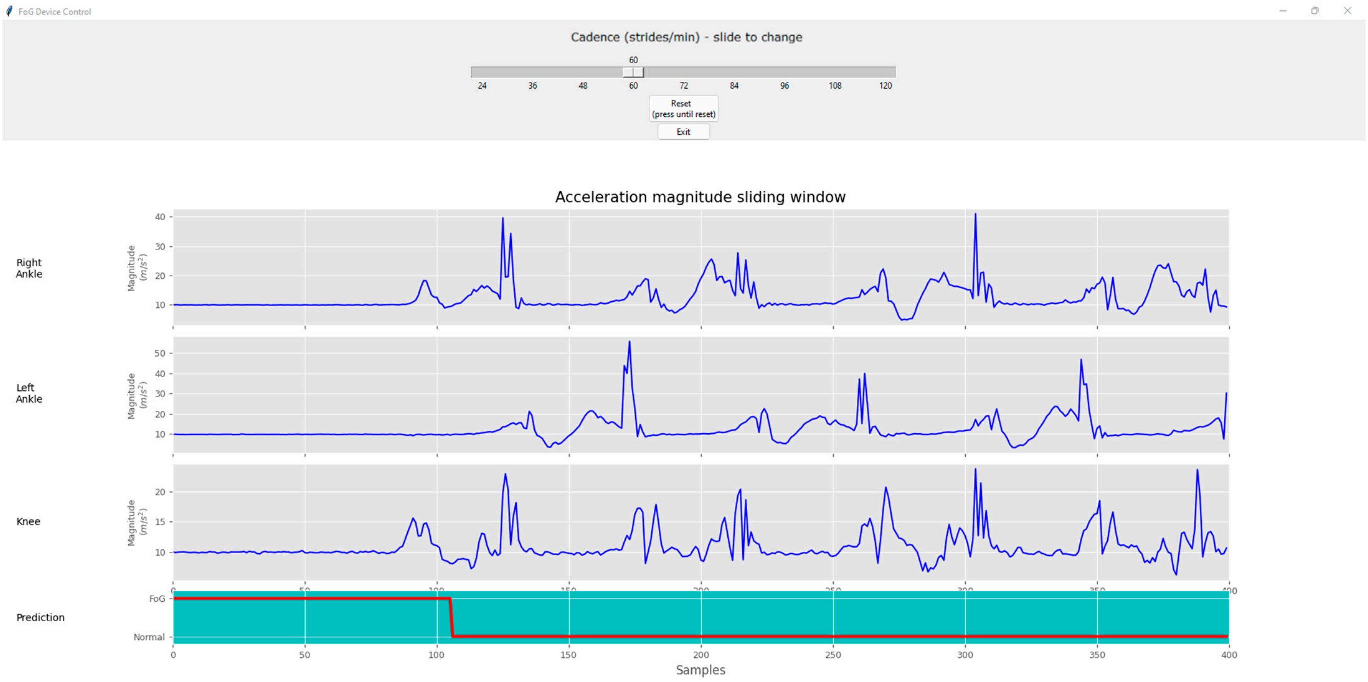

Figure S2 - Illustration of the developed graphical user interface.

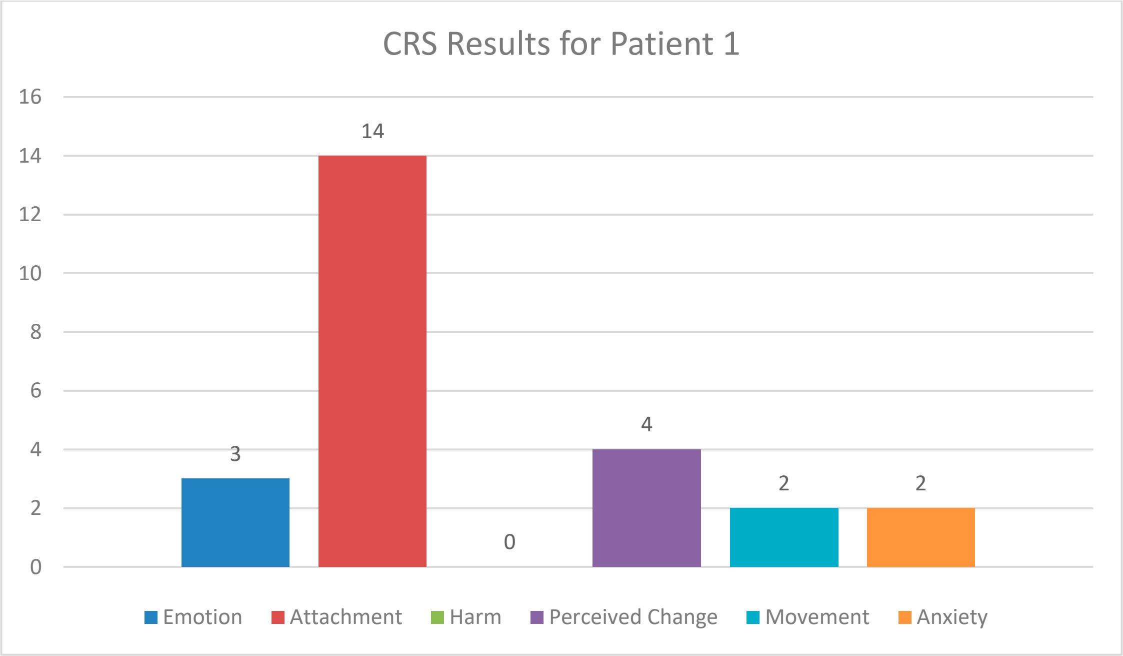

Figure S3 - Comfort assessment of the wearable device by patient 1. The scales used – CRS – range from 0 to 20, respectively, low and high.

2 Supplementary Tables

	Features	Description	Reasoning
Frequency-based Features	Highest Frequency Peak (HFP)	Frequency with highest power magnitude in the acceleration signal	Characterize stride
	Second Highest Frequency Peak (SHFP)	Frequency with second highest power magnitude in the acceleration signal	Characterize step
	Magnitude for Highest Frequency Peak (MHFP)	Power Magnitude of the highest frequency peak	Characterize stride
	Magnitude for Second Highest Frequency Peak (MSHFP)	Power Magnitude of the second highest frequency peak	Characterize step
	Freezing Index (FI)	Ratio of the power in the freezing band - 3 to 8 Hz - by the power in the locomotion band - 0.5 to 3 Hz	Tendency for power shift for higher frequencies during FoG

	Features	Description
Time-domain and Statistical Features	Min	Minimum of the signal
	Max	Maximum of the signal
	Median	Median value
	Mean	Average value
	Root Mean Square (RMS)	Quadratic mean value of the signal
	Standard Deviation (STD)	Mean deviation of the signal compared to the average
	Kurtosis	Degree of peakedness of the sensor signal distribution
	Skewness	Degree of asymmetry of the sensor signal distribution
	Entropy	Measure of the distribution of frequency components
	Zero Crossing Rate (ZCR)	Total number of times the signal changes from positive to negative or back, normalized by the window length
	Mean Crossing Rate (MCR)	Total number of times the signal changes from below average to above average, normalized by the window length
	Sum	Sum of magnitude over the entire window, normalized by the window length

Table S1 - Group of frequency-based features and time-domain and statistical features used in FoG detection algorithm their description and purpose in gait characterization.

Session	Stages	Videotape	Wearable (always recording IMU data)		Somatosensory stimulation			Auditive Stimulation-Metronome
					On- demand*			
			w/o vibrational motor	w/ vibrational motor	general algorithm	algorithm trained for each patient	Continuous	
1	1.1 Acquisition	X	X					
	1.2 Cueing w/ generic prediction	X		X	X			
2	2.1 Acquisition	X	X					
	2.2 Cueing w/ personalized prediction	X		X		X		
	2.3 Continuous Cueing	X		X			X	
	2.4 Auditive Cueing	X	X					X

*On-demand means that the stimulus is activated by the prediction model being only active during FoG episodes

Table S2 - Experimental study organization in two sessions with different stages. Motor protocol is repeated for all stages, each stage includes at least one variable change.

Test Patient	Specificity	Sensitivity	Precision	F1
1	73.1	90.1	13.6	23.7
2	74.7	100.0	35.5	52.4
3	81.8	69.1	36.5	47.7
5	79.5	72.3	49.5	58.7
6	80.7	84.3	22.5	35.5
7	73.2	95.9	12.4	22.0
8	77.1	69.5	50.4	58.4
9	79.8	69.7	36.7	48.1
Average	77.5	81.4	32.1	43.3
SD	2.9	11.2	12.0	12.2

Table S3 - Classification results of the testing set using the LOPO method for the eight patients of DAPHNet dataset that demonstrates freezing. The 'Test Patient' represents the set used to test the classifier trained with the remaining seven patients' data, using a SVM classifier.

Patient	Specificity	Sensitivity	Precision	F1	Prevalence
1	88.7	86.5	26.6	40.7	4.5
2	91.3	94.9	60.2	73.7	12.2
3	80.4	89.7	41.0	56.3	13.2
5	76.5	88.9	51.3	65.0	21.8
6	88.6	82.6	34.3	46.6	6.2
7	86.5	91.2	21.1	34.3	3.8
8	79.1	79.5	56.0	65.7	25.1
9	86.1	81.0	49.5	61.4	14.4
**Average**	84.6	86.8	42.5	55.5	12.6
**SD**	4.5	4.4	11.8	11.2	6.0

Table S4 - Classification results of the testing set for the eight patients of DAPHNet dataset that demonstrates freezing. The results were obtained through Repeated Stratified k-fold Cross Validation method for each patient using a SVM classifier. In addition to the metrics used to evaluate the classification, prevalence explains the ratio between the number of windows with freezing and the total number of signal windows. The values are in percentage.

	Patient 1	Patient 2
**How much the FoG symptom affects your daily life? (0 to 5 – no to high influence)**	4	4
**In what situations does FoG occur to you the most?**	Crowds, gait initiation	Crowds, gait initiation, overcome obstacles
**How many falls have you suffered due to FoG?**	1, no injuries	4, need medical attention
**Presents disabling sensory changes?**	No	Auditory only

Table S5 - Resume of questionnaire 1 important aspects.

3 Supplementary Notes

Note S1 - Detailed description of the DAPHNet dataset

The DAPHNet is a public domain dataset published in 2010 by Bächlin et al. [28] that has allowed the scientific community to accomplished advances in FoG detection. A total of 10 patients (3 women and 7 men) with PD participated in the experimental protocol that originated the DAPHNet dataset. They had an average age of 66.4 ± 4.8 years and disease duration of 13.7 ± 9.67 years. The average H&Y score was 2.6 ± 0.65. All tests were done in the OFF state (without medication that could mask FoG events) apart from 2 patients that experienced FoG events in the ON state. In Table S6 all the patients are characterized individually.

During data acquisitions, three accelerometer sensors were placed on each patient: in the ankle, thigh and lower back. The acquired data was transmitted via Bluetooth to the wearable computer at 64Hz. Each patient session took approximately 15 minutes and consisted of three different walking tasks: walking in a straight line, random walking, and activity of daily living (ADL).

Posteriorly, data was saved in a set of .txt files containing all the acquired raw data [acceleration (millig) of the ankle, thigh and lower back sensors in each of the 3 components (X, Y, Z)], timestamps for each datapoint and a manually chosen label for each type of observed event (0 - not part of experiment, 1 - normal or 2 - freeze). FoG episodes were visually identified by physiotherapists in a posterior analysis of video recordings. During the recording, patients 4 and 10 did not experienced any FoG episodes. The number of episodes detected range from 0 to 66 per patient culminating in a total of 237 FoG episodes. The length of episodes in this dataset ranged from 0.5 to 40.5 seconds.

Subject	Gender	Age (years)	Disease duration (years)	H&Y in ON	State during test
01	M	66	16	3	OFF
02	M	67	7	2	ON
03	M	59	30	2.5	OFF
04	M	62	3	3	OFF
05	M	75	6	2	OFF
06	F	63	22	2	OFF
07	M	66	2	2.5	OFF
08	F	68	18	4	ON
09	M	73	9	2	OFF
10	F	65	24	3	OFF
Mean ±STD		66.4 ±4.8	13.7 ±9.7	2.6 ±0.65	

Table S6 – Summary of subjects' information at the time of data acquisition for the DAPHNet dataset.

Note S2 - Detailed description of the signal processing pipeline for the DAPHNet data

The acceleration data – acquired from the inertial sensor – is sampled at a rate of 64Hz in each of the three axes. First, the mean value of each signal is removed, and the data are filtered through a 2nd order Butterworth low-pass filter with cut-off frequency of 15 Hz, removing high frequency noise artifacts. This step is helpful for frequency domain analyses since higher frequencies had no significance in detecting FoG according to the freezing band/normal band model by Moore (29, 30). Next, signal segmentation is performed, allowing to extract features representing a certain time window. Despite the segmentation being done after signal conditioning in the offline dataset, as usual, this is not possible in real-time detection. Therefore, during real time tests, signal conditioning is made every 0.5 seconds for the last 4 seconds of data. The Acceleration Magnitude (AM) is calculated before features are extracted.

$$AM = \sqrt{a_x^2 + a_y^2 + a_z^2}$$

Where a_x, a_y, a_z are, respectively, acceleration value in axis x, y and z.

Applying the AM ensures orientation invariance against displacements during user walking or eventual misposition of the sensor.

Note S3 - Questionnaires filled by volunteers in the trial

Sensor Vestível para Deteção Automática de Congelamento da Marcha em Doença de Parkinson – Questionário 1

Este questionário deve ser realizado após a primeira sessão de testes e para cada participante. O seu preenchimento é assistido pelo investigador que deve esclarecer possíveis dúvidas.

Informação sobre o participante

Nº______

Avaliação da marcha:

____

____

____

____

Notas:

____

Classifique o quanto o sintoma de congelamento de marcha afeta o seu dia-a-dia.

☐ 0 ☐ 1 ☐ 2 ☐ 3 ☐ 4 ☐ 5

Nada Muito

Em que situações lhe ocorre mais episódios de congelamento da marcha?

☐ Caminhar em linha reta ☐ Virar

☐ Atravessar portas ☐ Passagens apertadas

☐ Ultrapassar obstáculos ☐ Multidões

Iniciar marcha estando anteriormente:

☐ sentado ☐ deitado ☐ em pé

Outro: ____________________

Quantas quedas sofreu devido ao congelamento de marcha?

☐ 0 ☐ 1 ☐ <5 ☐ <10 ☐ <=20 ☐ >20

Se respondeu diferente de zero:

Classifique a queda mais grave que sofreu.

☐ Sem lesões ☐ Leve ☐ Moderada ☐ Grave

Apresenta alterações sensoriais incapacitantes a nível...

Visão? ☐ Sim | ☐ Não

Audição? ☐ Sim | ☐ Não

Tato? ☐ Sim | ☐ Não

Se apresenta alterações a nível do tato especifique.

____

Partilhe comentários ou sugestões adicionais.

____

____

____

____

____

____

____

____

____

____

Sensor Vestível para Deteção Automática de Congelamento da Marcha em Doença de Parkinson – Questionário 2

Este questionário deve ser realizado após a segunda sessão de testes e para cada participante. O seu preenchimento é assistido pelo investigador que deve esclarecer possíveis dúvidas.

Informação sobre o participante

Nº______

Avaliação da marcha:

____

____

____

____

Notas:

____

Está familiarizado com dispositivos de assistência ao congelamento de marcha?

☐ Sim | ☐ Não

Se sim, já usou diariamente algum dispositivo?

☐ Sim | ☐ Não

Qual?

____

Se sim, classifique o dispositivo usado (ou um deles-
_____________).

☐ 0 ☐ 1 ☐ 2 ☐ 3 ☐ 4 ☐ 5

Dececionante Excecional

Sente preconceito em usar um dispositivo visível?

☐ Sim | ☐ Não

Seria mais provável usar o dispositivo se este não fosse visível?

☐ Sim | ☑ Não

Sobre o dispositivo agora testado, considera prático o seu uso diário?

☑ 0 ☐ 1 ☐ 2 ☐ 3 ☐ 4 ☐ 5

Nada prático Muito prático

Classifique o dispositivo testado quanto ao conforto.

☐ 0 ☐ 1 ☐ 2 ☐ 3 ☐ 4 ☐ 5

Dececionante Excecional

Classifique a intensidade do estímulo sentido.

☐ 0 ☐ 1 ☐ 2 ☐ 3 ☐ 4 ☐ 5

Baixo Alto

Classifique o incómodo provocado pelo estímulo.

☐ 0 ☐ 1 ☐ 2 ☐ 3 ☐ 4 ☐ 5

Baixo Alto

Considera o estímulo suportável diariamente...

...em modo contínuo?

☐ Sim | ☐ Não

...só quando necessário?

☐ Sim | ☐ Não

Classifique a importância do estímulo estar ativo apenas quando necessário.

☐ 0 ☐ 1 ☐ 2 ☐ 3 ☐ 4 ☐ 5

Baixa Alta

Contando com eventuais falhas na deteção de episódios de congelamento e também de forma a preveni-los, considera importante existir um modo contínuo para situações mais suscetíveis de episódios de congelamento de marcha? (multidões)

☐ Sim | ☐ Não

Com efeitos comprovados, qual seria a probabilidade de adquirir o dispositivo?

☐ 0 ☐ 1 ☐ 2 ☐ 3 ☐ 4 ☐ 5

Nenhuma Alta

As próximas 6 questões dizem respeito à avaliação nas escalas de classificação de conforto (Comfort Rating Scales (CRS) – Knight et al. [1]).

Para cada uma das seguintes afirmações marque na escala com um 'X' o nível em que esta se aplica.

1. Emoção - Estou preocupado(a) como pareço quando uso este dispositivo. Sinto-me tenso ou nervoso porque estou a usar este dispositivo.

Baixo Alto

2. Fixação - Consigo sentir o dispositivo no meu corpo. Consigo senti-lo a mover-se.

Baixo Alto

3. Dano - O dispositivo está a provocar-me dano. É doloroso de usar.

Baixo Alto

4. Mudança percetível – Usar o dispositivo faz-me sentir fisicamente diferente. Sinto-me estranho(a) a usá-lo.

Baixo Alto

5. Movimento – O dispositivo afeta a forma como me mexo. Inibe ou restringe o meu movimento.

Baixo Alto

6. Ansiedade – Não me sinto seguro a usar o dispositivo.

Baixo Alto

Que valor estaria disposto a pagar pelo nosso dispositivo? (€)

☐ 0 ☐ <10 ☐ <20 ☐ <=50 ☐ >50

Partilhe comentários ou sugestões adicionais.

Note S4 – Discussion on feature selection

The Sequential Feature Selector was employed to determine the more relevant features to use in the classification algorithm by picking the 14 features that maximized the F1 score. The chosen features are grouped in an absolute frequency graphic in figure below. The frequency-based features, corresponding to the first five columns, were chosen in average 4.8 times and the time-domain features, were chosen in average 4.6 times. The least chosen features were the mean, RMS and the standard deviation, these three being the ones that will not be used. The feature corresponding to the sum of the signal from each window ('Sum') was used by the eight patients, which reveals its importance in the detection of FoG events. This is because, in general, the signal strength in the time domain is higher during normal gait than during freezing, in which the patient is moving less. Thus, the 'Sum' is a good indicator of FoG occurrence.

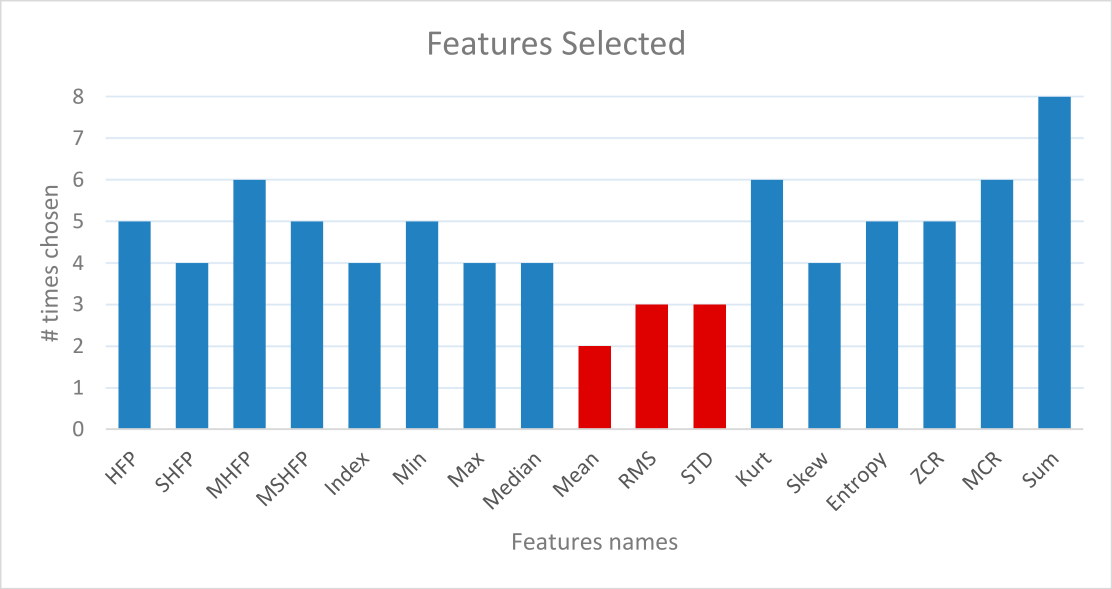

Figure S4 - Number of times that each feature was selected by the Sequential Feature Selector. Frequency-based features: HFP - Highest Frequency Peak, SHFP - Second Highest Frequency Peak, MHFP - Magnitude for Highest Frequency Peak, MSHFP - Magnitude for Second Highest Frequency Peak, Index - Freezing Index. Time-domain featured: Min – Minimum of magnitude signal, Max – Maximum of magnitude signal, Median, Mean, RMS – Root Mean Square, STD – Standard Deviation, Kurt- Kurtosis, Skew – Skewness, Entropy, ZCR – Zero Crossing Rate, MCR – Mean Crossing Rate, Sum – sum of magnitude values.
